# Computational meaningfulness as the source of beneficial cognitive biases

**DOI:** 10.3389/fpsyg.2023.1189704

**Published:** 2023-05-02

**Authors:** Jyrki Suomala, Janne Kauttonen

**Affiliations:** ^1^Department of NeuroLab, Laurea University of Applied Sciences, Vantaa, Finland; ^2^Competences, RDI and Digitalization, Haaga-Helia University of Applied Sciences, Helsinki, Finland

**Keywords:** confirmation bias, framing effect, computational meaningfulness, naturalistic stimuli, fMRI, machine learning

## Abstract

The human brain has evolved to solve the problems it encounters in multiple environments. In solving these challenges, it forms mental simulations about multidimensional information about the world. These processes produce context-dependent behaviors. The brain as overparameterized modeling organ is an evolutionary solution for producing behavior in a complex world. One of the most essential characteristics of living creatures is that they compute the values of information they receive from external and internal contexts. As a result of this computation, the creature can behave in optimal ways in each environment. Whereas most other living creatures compute almost exclusively biological values (e.g., how to get food), the human as a cultural creature computes meaningfulness from the perspective of one’s activity. The computational meaningfulness means the process of the human brain, with the help of which an individual tries to make the respective situation comprehensible to herself to know how to behave optimally. This paper challenges the bias-centric approach of behavioral economics by exploring different possibilities opened up by computational meaningfulness with insight into wider perspectives. We concentrate on *confirmation bias* and *framing effect* as behavioral economics examples of cognitive biases. We conclude that from the computational meaningfulness perspective of the brain, the use of these biases are indispensable property of an optimally designed computational system of what the human brain is like. From this perspective, cognitive biases can be rational under some conditions. Whereas the bias-centric approach relies on small-scale interpretable models which include only a few explanatory variables, the computational meaningfulness perspective emphasizes the behavioral models, which allow multiple variables in these models. People are used to working in multidimensional and varying environments. The human brain is at its best in such an environment and scientific study should increasingly take place in such situations simulating the real environment. By using naturalistic stimuli (e.g., videos and VR) we can create more realistic, life-like contexts for research purposes and analyze resulting data using machine learning algorithms. In this manner, we can better explain, understand and predict human behavior and choice in different contexts.

## Introduction

When making judgments or decisions, it is said that people often rely on simplified information processing strategies called heuristics, which may lead to systematic errors called cognitive biases (Berthet, 2021). Cognitive biases are considered human behaviors that violate normative standards of rationality from perspectives of classic logic and mathematics, described for example by the Expected Utility Theory (EUT; Von Neumann and Morgenstern, 2007). According to Gigerenzer (2018), the irrationality argument has become the backbone of behavioral economics. In this paper, we challenge such bias-centric approach to behavioral economics by exploring different possibilities by opening a wider perspective through the analysis of the phenomenon of computational meaningfulness.

It is a generally accepted idea that rationality is reasoning according to certain rules. Aristotle developed the logical syllogism and enthymeme as norms of human rationality. Logical syllogism links together a set of known premises to reach deductive conclusions, whereas enthymeme is suitable when a human has only limited knowledge about premises (Clayton, 2021). Furthermore, Descartes regarded the ability to use language during reasoning process as the hallmarks of rationality (Oaksford and Chater, 1994). However, most contemporary researchers emphasize, that rational rules should be described by rules of logic and mathematics. This idea of a rational decision-maker applying classical logic and mathematics is perhaps best described by EUT (Von Neumann and Morgenstern, 2007).

According to EUT, a rational decision-maker is a utility maximizer and s/he chooses the best option from those available (Kőszegi, 2010). Furthermore, EUT makes strong assumptions about rational decision-makers. First, they have stable and accurate representations of preferences and people respond to the options available to them independent of context and unaffected by other alternatives or temporal order (Suomala, 2020). Finally, a rational decision-maker behaves consistently and has all the necessary information to make a rational decision (Von Neumann and Morgenstern, 2007).

However, EUT produces predictions that are quite different from human behavior. It came under attack from researchers Tversky and Kahneman (1974) and Kahneman and Tversky (1979), who showed that humans cannot make rational decisions in the way that EUT and other normative theories had shown (Mckenzie, 2005). This BIAS-centric approach to BEHavioral Economics (BIASBEHA) has found a large number of cognitive biases and fallacies related to human choice (Tversky and Kahneman, 1974; Shafir and LeBoeuf, 2002; Ariely, 2009; Thaler, 2016). What the BIASBEHA has clearly shown is that the assumptions of the rationality of human behavior according to EUT do not have the power to explain, describe and predict human behavior in natural contexts. BIASBEHA has shown that people’s decision-making is predictably irrational because they use simple heuristics, which lead to systematic errors, or biases relative to EUT (Leonard, 2008; Ariely, 2009; Thaler, 2016).

When BIASBEHA has shown that a human’s decision-making does not follow the traditional principles of rationality, it falls into two serious fallacies. First, it does not take into account the complexity and flexibility of the human brain and real-life behavior with uncertainty. Behavioral research has traditionally been based on simplified models in which a certain behavioral phenomenon is explained by two or a few parameters. For example, Plato divided the mind into reason and emotion, and Descartes into the soul and body. Similarly, Kahneman (2011) follows Stanovich and West (2000), dividing thinking into system 1 (fast belief system) and system 2 (slow conscious and critical system). Although such simple divisions are fruitful metaphors for thinking, they are not capable of grasping the multidimensionality and flexibility of human thought. Second, it has mostly stripped the decision-maker of essential information—like prior beliefs—from its experimental setups. To move forward in the behavioral sciences, we should study people in those environments where they can use different sources of information in their behavior. We do not argue that BIASBEHA-approach has not any value in behavioral science. Of course, this tradition has increased our understanding of human behavior in different contexts. However, traditional experimental setups in psychology and other behavioral science are often too simple to capture the multidimensional human behavior and decision-making that takes place in different real-life contexts. We suggest that new neuroscientific and machine learning methods give new opportunities to provide an opportunity to bridge the gap between experimental research and real-life behavior (Jolly and Chang, 2019).

In this case, what is essential in a person’s behavior and decision-making is computational meaningfulness (Suomala, 2020; Suomala and Kauttonen, 2022), with which a person makes decisions in complex situations of everyday life. The computational meaningfulness approach assumes, that the brain/mind operates in different contexts by inquiring directly from the structure of the real world by optimizing multidimensional—with millions of parameters—information relating to the contexts. Previously, both the satisficing (Simon, 1955) and the bounded rational model (Gabaix et al., 2006) emphasize the study of human behavior in realistic and meaningful contexts. However, the model of computational meaningfulness takes into account the enormous parameter space of the brain, which is missing from the mentioned models.

According to the contextual approach to human behavior and decision-making, the task of the human brain/mind is to interpret the continuous complex information it encounters in a meaningful way in terms of one’s subjective goals and activities. There are thousands of potentially informative demographics-, dispositional-, personal-, genetic-, and neurobiological variables that correlate and affect human behavior. This process is inevitably very multidimensional and complex. Therefore, behavioral science needs tools to describe, explain and predict human behavior through models, which include hundreds or maybe thousands of parameters (variables; Yarkoni and Westfall, 2017; Jolly and Chang, 2019; Hasson et al., 2020). In addition, we describe the functioning of the human brain as a typical example of a biological computer processing huge information flows. The human brain’s basic processes are inductions and approximations and cognitive biases are a by-product of a process where the brain processes huge amounts of information utilizing induction and approximation. These are essential features of an optimally designed computing system, like the human brain.

With the recent development in machine learning and neuroscientific methodology as well as the increasing availability of large-scale datasets recording human behavior, we have good tools to understand better human behavior in real-life contexts (Yarkoni and Westfall, 2017). Therefore, from the computational meaningfulness perspective of the brain/mind, the use of cognitive biases may not be foolish at all and can be rational under some conditions (Gershman, 2021).

The article is organized as follows. We begin by describing typical assumptions of the BIASBEHA tradition. In addition, we describe more specifically cognitive heuristics relating to confirmation bias and framing effect. In conclusion of these, we highlight the problems relating to this tradition. Then, we describe a contextual approach with the recent development in machine learning and neuroscientific methodology. We end with our conclusions and suggestions on how to move forward BIASBEHA tradition.

## The heuristics and biases approach

The main aim of BIASBEHA was to study people’s beliefs about uncertainty and the extent to which they were compatible with the normative rules of EUT and other traditional logical calculus. This research program has been quite successful with thousands of scientific articles, Nobel laureates Daniel Kahneman and Richard Thaler in economics, and practical applications [e.g., Behavioral Insight Team in the United Kingdom government; popular non-fiction books: (Thaler and Sunstein, 2009; Kahneman, 2011)]. Moreover, new cognitive biases are constantly being discovered (Baron, 2008; Berthet, 2022), which give a rather pessimistic picture of human rationality. It is impossible to cover all these thinking biases in one article, so we will choose only two quite common and much-studied cognitive biases. These are the confirmation bias and framing effect. Below we describe typical example studies of both of them and the different interpretations made of them from the perspective of human rationality.

### Confirmation bias as an example of irrational human reasoning

The behavioral literature on how people should form and test hypotheses has borrowed heavily from the logic of scientific discovery. People tend to seek and interpret evidence in a way that supports their beliefs and opinions and reject information that contradicts them. This tendency has been regarded as confirmation bias (Nickerson, 1998; Austerweil and Griffiths, 2008; Gershman, 2021). The proclivity toward confirmation bias is considered one manifestation of people’s inability to think rationally (Wason, 1960, 1968; Popper, 2014). For example, Popper (2014) argued that science progresses through falsification, i.e., disconfirmation. A descriptive example of this is the discovery of helicobacter pylori.

In June 1979—on his 42nd birthday—Robin Warren saw something surprising with the new electron microscopy he had just adopted. A sample taken from the stomach of a patient with gastritis appeared to contain new types of curved bacteria. Although according to the bacteriology of that time, bacteria cannot live in the stomach because of its acidity, Robin Warren believed his eyes almost immediately (Warren, 2005). He was ready to disconfirm (i.e., falsify) the current theory of gastritis and started to find human and material resources, to make experiments to prove his observation correct (Thagard, 1998).

Despite strong opposition from his colleagues, he worked purposefully and decisively. Eventually, he was able to reform bacteriology with his colleague Barry Marshall related to the fight against diseases caused by helicobacter pylori in the stomach, and in 2005 they received the Nobel Prize in Medicine for their work (Warren, 2005).

Without a doubt, inventing something new is perhaps the highest degree of human mental ability and the clearest manifestation of human rationality. The cognitive-historical studies have shown that often scientific-, technological-and business breakthrough starts from unexpected perceptions (Suomala et al., 2006; Thagard, 2009). Warren’s case is a good example of this. The discovery of helicobacter pylori and demonstration of its effect in the development of gastritis and gastric ulcer is also a textbook example of the power of falsification in scientific discovery. The theory of bacteriology at the time was contradicted by Warren’s observation. Similarly, Galileo disconfirmed his time’s common theory that Moon has not any mountains. He made observations of mountains on the Moon with his new telescope and disconfirmed previous wrong theories. As Popper argued, science advances by falsification of current theories and hypotheses rather than by continually supporting theories (Popper, 2014). Typical for Warren’s and Marshall’s as well as Galileo’s case was that other scientists were against them and came up with several explanations with which they tried to save the old theories.

However, most ordinary people—like many scientists—do not apply disconfirmation as an inference strategy. Rather, they try to find support for their current knowledge and beliefs. The tendency to use confirmation means people’s proclivity to embrace information that supports their current beliefs and rejects information that contradicts them (Austerweil and Griffiths, 2008).

Illustrative examples of confirmation bias are attitude experiments about the death penalty (Lord et al., 1979) and the right to bear arms (Kahan et al., 2017). In the death penalty study, its supporters and opponents were asked to familiarize themselves with two fictional empirical studies. Individuals who supported capital punishment subsequently strengthened their belief in the effectiveness of the death penalty after reading the two studies, whereas individuals who opposed capital punishment subsequently strengthened their beliefs in its ineffectiveness. The conclusion of the effect of the data evaluations is that opinion shifts of the participants increase attitude polarization (Lord et al., 1979; Gershman, 2021). The same body of evidence confirms people’s individual beliefs in opposite directions indicating humans’ tendency to confirmation bias.

While the content of the study of Lord et al. (1979) above was a complex and emotional social issue, does the effect of confirmation bias decrease, when the content is not so emotionally charged content? The attitude study about the right to bear arms (Kahan et al., 2017) tackled this question. In the study, the participants were presented with a difficult problem that required numeracy—a measure of the ability to make use of quantitative information. As expected, participants highest in numeracy did to a great extent better than less numerate ones when the data were presented as results from a study of a new skin-rash treatment. However, when the content of the inference changed from fact-based to emotionally charged content, the situation changed. Now, the participants evaluated the results from the fictional study of a gun-control ban. Now subjects’ responses became less accurate and politically polarized. Such polarization did not abate among subjects highest in numeracy, rather, people who were good at numeracy used their talent to strengthen their own beliefs similarly to people with lower numeracy.

The rule learning task of Wason (1960) and selection task of Wason (1968) are the most cited examples relating to confirmation bias. Human reasoning in these tasks has been considered an apt exemplification of human irrationality. In the rule learning task, participants need to generate triples of numbers to figure out what the experimenter has in mind. This task is a more demanding version of the generally known object recognition task with 20 questions (Navarro and Perfors, 2011). The allowable queries in both queries are in the general form “Does x satisfy the rule?,” where x is an object in 20 question game and a number in Wason’s rule learning game (Navarro and Perfors, 2011). Wason gave the triple “2-4-6” as an example of the rule. Then the participants were asked to construct a rule that applies to a series of triples of numbers to test their assumptions about the rule the experimenter had in mind. For every three numbers the subjects will be coming up with, the experimenter will tell them whether it satisfies the rule or not, until the subject comes up with the right rule (Wason, 1960).

Most participants first formed a hypothesis about the rule: a sequence of even numbers. Then they tested this rule by proposing more sequences of numbers typically “4-8-10,” “6-8-12,” and “20-22-24.” The feedbacks to all these sequences were positive. The participants produced a few more tries until they felt sure they have already discovered the rule. Most participants did not discover the rule, which was simply “increasing numbers.” Wason (1960) showed that most of the participants avoided falsifying their hypotheses and instead sought to find confirmation for their hypotheses.

In the selection task (Wason, 1968), participants are presented with four cards (A, K, 2, and 7), each with a number on one side and a letter on the other, and a rule “If a card has a vowel on one side, then it has even number on the other side.” Thus, the rule has a general form “if p, then q.” Participants have to select those cards that they must turn over to infer whether the rule is true or false. Following the argument of Popper (2014) about falsification (disconfirmation), the correct choice is to turn over the vowel card (A) and the odd card (7) because finding an odd number behind the vowel or a vowel behind the odd number would reveal the hypothesis to be false. In other words, according to Popperian rationally, the correct answer follows a falsificationist (i.e., disconfirmation) strategy. It appeared that only 4% of subjects used the disconfirmation strategy. By contrast, the vast majority of participants used the confirmation strategy by either only turning over the vowel card (A; 33%) or turning over the vowel (A) and even cards (2; 46%). In other words, people seem to be following a confirmation test strategy, turning over cards that confirm the rule.

The studies described above regarding confirmation bias have been taken as strong evidence that humans are fundamentally irrational in their reasoning. This shows up as an irrational belief updating of individuals (Kunda, 1990; Gershman, 2021) and a strong tendency to strong logical errors in individuals reasoning (Wason, 1960, 1968; Johnson-Laird and Wason, 1970; Kahneman, 2011; Thaler, 2016). These experiments show that participants violated Popper’s normative rule, according to which a rational actor pays attention to things that contradict the reasoner’s presuppositions. Instead, participants tested their hypotheses in a way that would lead them to be confirmed. We as humans gather information in a manner that leads us to believe or to strengthen our subjective presuppositions regardless of their correctness.

### Confirmation bias as an example of the adaptability of human reasoning

However, wider interpretations of the phenomena of confirmation bias have been presented (Oaksford and Chater, 1994; Mckenzie, 2005; Navarro and Perfors, 2011; Gershman, 2021). In addition, many philosophers of science have rejected falsificationism as unfaithful to the history of science and to be anyway unworkable (Lakatos, 1970; Kuhn, 1996; Churchland, 2002). These new interpretations emphasize that confirmation bias can be rational under some conditions (Gershman, 2021). We present some of them below.

According to this broader view, when a person acts in a certain situation, the person tries to grasp those environmental cues that increase his/her understanding of this situation. Especially, the interpretation of an event is an inferential process and during this process, an individual tries to increase knowledge and decrease uncertainty. In this case, the confirmation approach can be the most effective strategy.

Whereas Warren’s and Marshall’s discovery of helicobacter pylori is a good example of Popper’s understanding of scientific discovery (Popper, 2014); science progresses by falsification. However, there are also contrasting examples in the history of science. When astronomers discovered Uranus in 1781 and noticed that it was deviating from its predicted orbit, they did not try to disconfirm the prevailing Newtonian theory of gravitation (Clayton, 2021; Gershman, 2021). Thus, they behaved in similar ways as participants in Wason’s experiments. They persistently sought a Newtonian-compatible explanation for Uranus’ unusual trajectory and Le Verrier and Adams in 1845 independently completed calculations showing that the unusual trajectory of Uranus could be entirely explained by the gravity of a previously unobserved planetary body (See Gershman, 2021). Eventually, a year later Johann Gottfried Galle found through telescopic observation Neptune in the night sky almost exactly where Le Verrier and Adams predicted it had to be. These astronomers succeeded in two ways: they discovered a new planet, and they rescued the Newtonian theory from disconfirmation (Gershman, 2019).

Moreover, contemporary research has argued that belief polarization might arise from different auxiliary hypotheses about the data-generating process (Jaynes, 2003; Jern et al., 2014; Cook and Lewandowsky, 2016; Gershman, 2019). The mental simulations of people’s brains do not include perfect natural, mental, and cultural events. As Gershman (2021) argues, resistance to disconfirmation can arise from the rational belief updating process, provided that an individual’s intuitive theories include a strong prior belief in the central hypothesis, coupled with an inductive bias (Suomala and Kauttonen, 2022) to posit auxiliary hypotheses that place a high probability on observed anomalies. Jern et al. (2014) explained the findings of Lord et al. (1979) by using a rational Bayesian framework. When subjects in the experiment do not trust the results of the research, then reading a report about the ineffectiveness of capital punishment may strengthen their belief. These beliefs in research bias could include doubt about the validity of the experimenter, data source of stimuli, and other auxiliary arguments against the evidence presented during experiments as a whole (Corner et al., 2010). Similarly, Cook and Lewandowsky (2016) demonstrated that belief polarization and contrary updating are consistent with a normative rational approach using the Bayesian framework. Thus, various auxiliary hypotheses are almost always in play when a human makes inferences. When one’s beliefs about auxiliary hypotheses will change, then the interpretation of observations will also change (Gershman, 2021). Next, we will look at the new interpretations of the results of Wason’s tasks.

Several researchers consider that the structure of Wason’s tasks is such that it favors the confirmation strategy in reasoning. Klayman and Ha (1987) found that confirmation bias can be understood as resulting from a basic hypothesis-testing heuristic, which they call the Positive Test Strategy (PTS). According to PST, people tend to look at instances where the target property is assumed to be present. Klayman and Ha (1987) emphasized that most task environments are probabilistic and then it is not necessarily the case that falsification provides more information than verification. What is the best strategy depends on the characteristics of the specific problem at hand.

For example, the true rule in the rule learning task, which the experimented has in mind (“increasing numbers”) is more general than the tentative plausible hypotheses in participants’ minds (“increasing intervals of two”; typically “4-8-10″, “6-8-12″, “20-22-24″). In this case, people tend to test those cases that have the best chance of verifying current beliefs rather than those that have the best chance of falsifying them (Klayman and Ha, 1987). Furthermore, PTS is more likely when testing cases people expect will not work to lead to disconfirmation when people are trying to predict a minority phenomenon (Klayman and Ha, 1987; Mckenzie, 2005). These two conditions are commonly met in real-world reasoning situations and the confirmation strategy appears to be the rational strategy during reasoning.

Furthermore, Oaksford and Chater (1994) argue that turning the A and the 2 cards (confirmation) in Wason’s card selection task is the most informative for determining if the rule is true or not. The confirmation strategy epitomizes general findings that rare events are more informative than common events (Klayman and Ha, 1987; Mckenzie, 2005). Thus people infer that the rule includes rare items—as vowels in English are—then the PTS shows the rational approach to the task contrary to Wason’s interpretations and many other researchers’ interpretations (Wason, 1960, 1968; Johnson-Laird and Wason, 1970; Kahneman, 2011; Thaler, 2016).

A descriptive example of the human ability for adaptable reasoning is manifested in a version of the game “Battleship” (Hendrickson et al., 2016). The game took place on a 20 by 20 grid partially covered by 5 ships (gray rectangles). The task of participants in this game is to discover the correct arrangement of the ships in the grid. They could ask where the ships were located (confirmation strategy) or where they were not located (disconfirmation strategy). Participants were told that their goal was to position the ships in their correct positions. The correct positions were randomly selected from a large set of possible configurations (Hendrickson et al., 2016). Participants were randomly assigned to one experimental condition in which the size of the ships was manipulated such that the portion of the grid covered by the ships ranged from 10% to 90%. In small ship conditions, there were many more legal candidate hypotheses than in large ship conditions since there were many more possibilities in which no ships overlapped in small ship conditions (Hendrickson et al., 2016).

The research demonstrated that there is a clear relationship between hypothesis size (i.e., legal potential position) and the degree to which people prefer confirmation strategy. In the 10% condition the average preference for confirmation strategy (i.e., questions, where the ships are located) was 86%, whereas, in the 90% condition, it was only 36%. Consistent with optimal information-acquisition strategy, when the size of ships increased (i.e., legal potential positions decreased), the confirmation request declined. The study showed that the request for positive evidence (confirmation) declined as the size of hypotheses (literally the size of ships) increased, consistent with the optimal information-acquisition strategy.

When the findings of confirmation biases have been regarded as a manifestation of irrational human behavior, contemporary research—as we described above—has shown that this traditional approach is too narrow. Preference for confirmation reflects the structure of how people represent the world (Gershman, 2021). The ability to adapt, to act actively and flexibly in different environments is an indication of human rationality, although can sometimes lead to preposterous beliefs. Now we concentrate on other cognitive biases presented in heuristics and bias tradition, namely the framing effect.

### Framing effect as an example of irrational human reasoning

The framing effect occurs when people’s choices systematically depend more on how the information of objects or outcomes is described than the substance of the pertinent information (Mckenzie, 2005; Leong et al., 2017). It is considered cognitive bias because an individual’s choice from a set of options is influenced more by how the information is worded than by the information itself.

In attribute framing tasks one frame is usually positive and one negative (Levin et al., 1998). Ground beef is evaluated as better tasting and less greasy among participants when it is described in a positive frame (75% lean) rather than in a negative frame (25% fat; Levin and Gaeth, 1988). Similarly, when a basketball player’s performance is described in terms of performance of shots “made” (positive frame) rather than “missed” (negative frame), participants rate the player as better in terms of abilities in positive than negative condition (Müller-Trede et al., 2015).

Furthermore, the attribute framing effect is found in contexts of plea bargaining (Bibas, 2004) and among economists (Gächter et al., 2009). The analysis of plea-bargaining literature has brought up the effect of framing on the criminal justice system (Bibas, 2004). The effect of framing appears to be a crucial component in the process, although skillful lawyering may ameliorate its effect. Similarly, the framing effect of conference payment for the participants of a scientific conference for behavioral economics has been studied (Gächter et al., 2009). The results showed that while the junior experimental economics was influenced by the framing effect, the more senior economists were not (Gächter et al., 2009). In a similar vein, people who are knowledgeable about an attribute’s distribution (i.e., what is the typical number of free throws scored per season by an athlete playing basketball in the NBA) exhibited a reduced framing effect in the basketball framing scenario. However, the framing effect was unaltered among the same people in the medical framing scenario, of which they had no prior knowledge (Leong et al., 2017).

It is worth noticing that the information framed above examples is not the outcome of a risky choice but an attribute or characteristic of the goods. However, the best-known examples of framing effects involve choosing between a risky and a riskless option that is described in terms of either gains or losses (Kahneman and Tversky, 1979, 1984; Tversky and Kahneman, 1981). When the options are framed as risk-level, gains, and losses, the reference point has an important role. Moreover, people are more willing to take risks when the information is framed negatively but seek to avoid risks when the information is framed positively (Tversky and Kahneman, 1981).

According to Prospect Theory (Kahneman and Tversky, 1979), a decision maker transforms objective values of offers to subjective values at the present of the reference point according to the S-shaped value function. In this case, a human feels the loss relatively stronger than the gain about a reference point. At first, the Prospect Theory has described human choice in contexts, where a decision maker’s status quo at the time of each choice dictates the subjective reference point (Kahneman, 2003). In these situations, a decision maker perceives any negative departure from her status quo as a loss, while perceiving any positive departure from the same status quo as a gain (Tversky and Kahneman, 1981; Louie and De Martino, 2014). Later, there is growing evidence that people evaluate the outcomes in light of the expectations or their subjective goals which act as a reference point, similar to the status quo as a reference point (Camerer et al., 1997; Heath et al., 1999; Koszegi and Rabin, 2006; Abeler et al., 2011; Suomala et al., 2017). Therefore, the prospect theory is crucial to understanding the framing effect. It describes how people evaluate their losses and acquire insight asymmetrically.

This phenomenon is aptly described in the famous Asian disease-study (Tversky and Kahneman, 1981). In the study, the participants were asked to choose between two options for treatment for 600 people, who suffer from a dangerous imagined Asian disease. The first treatment was likely to result in the deaths of 400 people, whereas the second treatment had a 66% possibility of everyone dying and a 33% possibility of no one dying. These two treatments were then described to the participants of the experiment with either a negative framing (describing how many would die) or a positive framing (relating how many would live). The result of the study (Tversky and Kahneman, 1981) showed that 72% of participants chose the first option for treatment when it was framed positively, i.e., as saving 200 lives. However, only 22% of participants chose the same option when it was framed negatively, i.e., resulting in the deaths of 400 people. Similarly, when survival rates of a surgery or other medical procedure are emphasized, people are more likely to approve of the procedure than when the mortality rates of the procedure are emphasized (Levin et al., 1998).

Despite there being some evidence that the framing effect was attenuated for those participants knowledgeable about the context (Gächter et al., 2009; Leong et al., 2017), it is widely considered to provide clear-cut evidence of irrationality and systematic violations of the axioms of rationality in decision-making in the same way as the confirmation bias (Kahneman and Tversky, 1979; Kahneman, 2011). Framing effect violates especially the description invariance-principle (Von Neumann and Morgenstern, 2007) essential normative principle in EUT (Mckenzie, 2005). However, recent studies—as we described below—have shown that this is not necessarily the case.

### Framing effects as an example of the adaptability of human reasoning

Recent studies related to human behavior have shown, that humans and other mammals are sensitive to the context as a whole (Gallistel and Matzel, 2013; Müller-Trede et al., 2015). The context as a whole has often a stronger effect on behavior than single objects or objects’ attributes. Even when participants process information about artificial objects (i.e., stimuli) in decontextualized experiments, participants have a proclivity to form rich and versatile mental simulations, which include not only the stimuli but also the likely context and its latent causes in which these stimuli typically occur (Gershman et al., 2015; McKenzie et al., 2018; Cushman and Gershman, 2019). In these experimental as well as in real-life contexts, an individual infers based on her/his prior experience and expectation relating to a context as a whole (Baum, 2004; Gershman and Niv, 2013; Suomala, 2020; Suomala and Kauttonen, 2022). For example, when the above-described task includes the wording “the ground beef is 75% lean,” a participant likely tries to understand this wording from the point of view of either the experimenter or the butcher (Leong et al., 2017). Then this context leaks information about the experimenter’s and the butcher’s intentions, and these informative signals are different in different options, despite options being logically equivalent (McKenzie and Nelson, 2003; Suomala, 2020).

Each real-life context contains an almost infinite number of configurations in terms of human interpretation ability. The human resolves this problem of abundant information flows by utilizing prior experiences (i.e., memories) and contextual information. When a researcher constructs the experiment, the narrative, and single words form the information context for participants. Mckenzie (2005) and Sher and McKenzie (2006) argues that the frame chosen by the researcher and its linguistic expression constitute the information content for the test subjects with reference points chosen by the researcher. In these cases, logically equivalent frames can signal relevant information beyond the chosen frame’s literal content. For example, McKenzie and Nelson (2003) found that the “speaker” participants were more likely to express a cup with liquid at the halfway mark as “half empty” rather than “half full” when the cup had initially been full and was therefore empty. Then “Listener” participants, in turn, “absorbed” the information signaled by the speaker’s choice of frame and were more likely to infer that a cup was originally full when it was described as “half empty.” In other words, listeners’ inferred reference points matched the actual reference points that guide speakers’ frame selection. McKenzie and Nelson (2003) conclude that logically equivalent frames can often implicitly convey different information and participants are sensitive to this different information. Then logically equivalent frames can convey choice-relevant information and participants in the experiments exploit this information effectively (McKenzie and Nelson, 2003; Sher and McKenzie, 2006).

Human behavior from sensory observation to mental simulation constructions is guided by the principle of meaningfulness (Suomala, 2020; Suomala and Kauttonen, 2022; Gershman, 2023). This sense-making process emphasizes certain features of the context at the expense of other features. The human brain integrates incoming extrinsic information with prior intrinsic information to form rich, context-dependent models of situations as they unfold over time (Yeshurun et al., 2021). How individuals can weigh different elements when constructing the important elements of the context? An illuminating example is the study (Sher and McKenzie, 2014), which provided experiments, where the participants were asked to evaluate a suitable salary for coders and buy CDs.

In the salary experiment, participants saw three things about two applicants. Both had graduated from the University of San Diego with majors in programming. The average grade of Applicant A was 3.8 (max 4.0) and Applicant B was 3.1. In addition, A had programmed 10 programs in the YT programming language, while B had programmed 70 programs in the same language. The essential point here is that knowledge relating to the University of San Diego and grade were familiar to the participants, whereas the YT programming language was unknown to them. The participant groups, which evaluate individual applicants, based their evaluation on the known attributes. In this case, A applicant got a better salary suggestion than B applicant. This is understandable because the A applicant was better in grade than the B applicant. These individual evaluation groups ignored the effect of programming experience because they likely did not understand its meaning. However, the third group of participants evaluated both A and B applicants’ salaries at the same time. In this case, participants suggested better salaries for B applicants. Despite the YT programming language being unknown among participants in this group, they were likely sensitive the relatively large difference (10 programs vs. 70 programs) between applicants.

Similarly, in CD study, participants showed their willingness to pay for different CD boxes. When individual CD-box was presented, unknown attributes were ignored by participants. However, when different versions of CD-boxes were presented at the same time, participants were capable to evaluate different versions and they also interpret unknown attributes of each other to make suitable price estimates (Sher and McKenzie, 2014). Thus, people are very sensitive to both implicit and explicit contextual clues, when trying to make sense of the context.

It is possible to assume, that the researchers planning an experiment form specific frames and reference points, and these original choices affect test subjects’ inference processes about these frames. For example, the medical tasks described above illustrate that describing the treatment in terms of percent survival signals that the treatment is relatively successful, whereas describing it in terms of percent mortality signals that the treatment is relatively unsuccessful. This speaker–listener interpretation help explain also people’s behavior in other framing contexts, which we described above.

The speaker-listener framework is reminiscent of Gricean notion of conversational implicature (Grice, 1975; Corner et al., 2010). According to conversational implicature, information is not contained in the literal content of an utterance but can be implied from the context in which it is given (Grice, 1975). Corner et al. (2010) emphasized that participants may infer more about the experiment than is contained in the literal content of the instructions and participants might have different ideas about what key task parameters are—such as the diagnosticity of evidence in belief revision experiments.

Similarly, people try to interpret the content of information based on plausibility (Jaynes, 2003). For example, in the case of Asian disease (Tversky and Kahneman, 1981) described above, it is very difficult to imagine that such a treatment would exist in real life. Recent research (Cohen et al., 2017) on the ability to reason in medical cases showed, that people’s inference is rational in the traditional sense when the probabilities were believable. Similar logically consistent reasoning has been observed in syllogistic reasoning, where beliefs about the plausibility of statements based on everyday experience influence truth judgments (Revlin et al., 1980). Jaynes (2003) emphasizes that people’s inference is neither deductive nor inductive, but it is plausible reasoning. It has strong convincing power, and a human decides this way all the time (Suomala and Kauttonen, 2022). Thus, people’s reasoning process is not necessarily purely syntactic or computational. Rather, it is sensitive to meaningful properties of the combination formed by observation and prior experience. When the occurrence of objects and their frames and their relationships are meaningful from an individual perspective, her/his reasoning process appears to be rational (Gershman, 2021).

Above we have described examples of heuristic and biased approaches to the confirmation bias and the framing effect. Results in these studies appear to show that people do not reason according to the principles of classical rationality. In both confirmation effect—and framing effect experiments people’s performance appears biased when compared with the standards of logic, probability theory, and EUT. However, contemporary critical studies showed that the human mind is more flexible, context-sensitive, and capable to interpret environmental features based on an individual’s prior experiences. These studies considered misleading the purely negative view of human performance implied by the BIASBEHA approach.

Despite the current new critical approach to heuristics and biases, tradition has taken important steps in contextualizing human behavior, we must go further. As most of the empirical studies of human behavior—also these critical studies—suffer from the flatland fallacy (Jolly and Chang, 2019).

Term Flatland fallacy refers to Edwin Abbott’s famous Novella Flatland: a Romance of Many Dimensions (Abbott, 2019), in which the creatures (Flatlanders) with limited perceptual capacities (i.e., seeing in only two dimensions) come to reason in a limited way. They ignored the complexity of the world and believed that their perceptions are veridical. Jolly and Chang (2019) argued that much like Flatlanders, humans exhibit strong biases in their reasoning about a complex and high-dimensional world due to finite limitations on their cognitive capacities. They claim that most psychological researchers are like Flatlanders and try to understand human behavior with impoverished models of human behavior. We agree and suggest that most of the results of BIASBEHA-tradition are a result of not taking the multidimensionality of human behavior into account. To overcome this fallacy, we should study human behavior under as natural conditions as possible. In the following chapters, we describe this approach more specifically.

## Computational meaningfulness as the core of the human rationality

To move forward in the behavioral sciences, it is central to understand the behavior of people in real-life contexts. Our mind is not a photocopier. Rather it is a biological computer that extracts meaningful patterns from contexts to know how to behave adaptively in each context (Suomala, 2020). In this chapter, we describe factors that, according to our understanding, help behavioral scientists to conduct better research that takes into account human operating naturalistic environments. At first, we need a theoretical model of human behavior. Such a model should include the following factors (Hofstadter, 1979; Gallistel, 2009):

1. It realistically describes the signals that humans process, and how those signals are processed to yield action.
2. It realistically identifies meaningful actions.
3. Research results increase our understanding of human behavior in natural environments.

We claim that BIASBEHA approach does not include the three factors listed above. Next, we describe the foundation for a new behavior model based on the criteria described above.

### The signals that humans process

Living creatures, from single-celled organisms to humans, always function in a certain context (Suomala, 2020). For a human, these contexts are usually cultural environments, the meanings of which a growing child learns to understand. When behaving in a certain context, a person computes information from the context to serve her activities. We call this process of transformation and utilization computation. Computation means the process by which the human brain transforms the contextual information and combines these with mental simulations previously adopted by the individual in order to behave in optimal ways (Tegmark, 2017; Suomala and Kauttonen, 2022).

This means that a person always develops, learns, and acts in a certain cultural context. This is aptly illustrated by the study (DeCasper and Spence, 1986) that showed that a child learned to prefer the fairy tale “The Cat in the Hat” during the fetal period, which one’s mother read regularly at the end of the waiting period. Thus, children’s preferences begin to be biased toward certain cultural things—in this case specific fairy tales—that are present in their environments. In other words, a child begins to embrace important cultural entities and to behave in this specific cultural context adaptable. Whereas the early learning of a child is likely limited to reasoning about objects and agents in their immediate vicinity, the wider cultural artifacts, values, and habits develop later with interactions of the child and other people and official institutions. During this process, the most crucial aspect of the human mind is the motivation to share culturally meaningful aspects with others (Tomasello et al., 2005; Tomasello, 2014; Suomala and Kauttonen, 2022). So, the contexts include not only the physical objects but above all the cultural entities. These contexts offer a person potential behavioral opportunities, which we call cultural affordances. A person learns and acquires knowledge and skills and may develop into an expert in some field. Growing into an expert is situational in nature.

Humans process signals from their contexts, which include constellations of cultural affordances. Described in this way, the concept of cultural affordances is related to Gibson’s concept of affordance (Gibson, 1979) and Hasson’s direct-fit approach (Hasson et al., 2020). The human brain constructs continuous experiences about the world to behave in optimal ways in a specific context. The real-life contexts in our society are complex, dynamic and uncertain, containing typically “countless” numbers of objects, the path of objects, people, and their interactions.

Thus, the world—physical and cultural—around us includes an almost infinite amount of information from a human point of view. The human resolves this problem of abundant information flows by using prior experiences (i.e., memories) and contextual information. In other words, from the point of view of humans, the world contains much more potential information than one can convert into knowledge according to her/his purposes.

The human brain computes the meaningful constellations about the contexts. It can extract meaningful patterns from complex and information-rich environments because the human brain has evolved specifically to function in complex and uncertain contexts. Despite the absolute number of neurons in the human brain remaining unknown, the approximation is that it has about 85 billion neurons (Azevedo et al., 2009) and it is each cubic millimeter contains roughly 50,000 neurons. Because these neurons may support approximately 6,000 adjustable synapses with other cells, this structure yields about 300 million parameters in each cubic millimeter of the cortex and over 100 trillion adjustable synapses across the entire brain (Azevedo et al., 2009; Hasson et al., 2020). Thus the human brain is overparameterized organ and it can produce flexible, adaptive behavior in a complex world (Hasson et al., 2020).

Even though the brain is efficient, an individual is only able to compute a small part of the information in the context with it. Let us imagine a six-year-old child buying penny candies with 10 different candies. The child is allowed to choose 10 candies. Mathematically, and following the rules of EUT, 10 different candy combinations in this context can form 92,378 different options. If it took 15 s to collect one bag, it would take a child a good 384 h, or a good 16 days, to try all these candy combinations if she did nothing else during that time. However, in real life, she can choose candies in a few minutes. We all make this kind of decision daily and despite the department store including over 100,000 items, we rarely spend more than an hour there. We do not behave according to EUT (Bossaerts and Murawski, 2017).

In a conclusion, people process only part of potential signals in a context. People are developed and learned to see easily things that our culture hands us ready-made as cultural affordances in different contexts (Hofstadter, 2001; Zadbood et al., 2021). If these meaningful constellations are lacking—like in typical BIASBEHA experiments—people still try to interpret minor context clues to make them understandable to themselves. This leads to false conclusions about behaviors that do not align with those made in real life.

People learn most frequently encountered cultural constellations over a lifetime. The learned constellations are stored in long-term memory as multidimensional and dynamic experiences. We call these stored memories as mental simulations because these memories are more vivid and dynamic movies than static object-like properties (Barsalou, 2009). Through these learned constellations, the past is intertwined with a person’s present and future (Gallistel, 2017). Mental simulations of the contexts in the brain are dynamics networks where context-related information is stored in nodes. The links are synapses that carry messages from nodes to other nodes.

### The meaningful actions as the human represent it

Like the contexts surrounding the individual, the mental simulations relating to the contexts stored in the individual’s brain are also “countless.” An individual has constructed them of experienced contexts during her/his lifetime. These context-based simulations are strongly domain-specific and intuitive. These mental simulations support an individual to produce flexible and meaningful behavior in a complex world.

The meaning of a context and meaningful actions are formed by the weights of individual nodes and their links to other elements of the context (and between contexts) in the brain (Hofstadter, 2001; Yeshurun et al., 2021). This forms a graph where context and actions are interconnected, not independent from each other. In other words, the elements of mental simulations, which need more memory resources, are more meaningful for a subject than elements that need just a few resources.

The objects, other people, cultural artifacts, and conventions and their interactions happen in specific contexts, and humans learn to behave in these contexts gradually. We are not born with an understanding of entities and their roles in specific contexts. This understanding must be learned from experience. As a child grows up, one’s starts to perceive constellations of events. Then a growing child begins to construct fragments from life’s streams as constellations as high-level wholes (Hofstadter, 2001). These learned complex constellations are constructed based on the principle of computational meaningfulness. This principle means, that the human brain can produce a set of constraints concerning the distinction between different constellations (a bunch of stimuli) of cultural affordances. Thus, computational meaningfulness is the result of human’s ability to differentiate constellations from one another on a given set of observations. To do that, humans need the mental resources to choose the most meaningful features of the environment to behave in optimal ways in this environment (Ratneshwar et al., 1987; Suomala, 2020; Suomala and Kauttonen, 2022). In this way, a person learns to extract important aspects of the experienced context (Gallistel and Matzel, 2013).

Since each real-life situation contains an almost infinite number of possible configurations in terms of human interpretation ability, the human ability to assign meanings to certain constellations at the expense of others can be considered rational behavior (Hofstadter, 1979, 2001; Ratneshwar et al., 1987; Suomala, 2020).

Above we described the properties of contexts, the human brain, and mental simulations. When an individual acts in the context, s/he tries to find meaningful constellations about the current context and tries to figure out, how these constellations support her/his personal goals. How do these comprehensive processes and the human ability to find meaningful constellations in different contexts manifest human rationality?

Computational meaningfulness means the process of the human brain, with the help of which an individual tries to make the respective situation comprehensible to herself to know how to behave optimally in a specific context. Then rationality means four things. First, it means that the brain makes different contexts understandable by inquiring directly from the structure of the real world by recognizing the relative importance of different elements in these contexts by optimizing multidimensional—with millions of parameters—information relating to these contexts (Hofstadter, 1979; Hasson et al., 2020). Second, it means that a human can respond to contexts very flexibly and can make sense of ambiguous or contradictory messages (Hofstadter, 1979; Geary, 2005; Gershman, 2021). Third, it means that an individual can set complex goals and finally, it means that an individual can achieve these goals (Geary, 2005; Tegmark, 2017). In summary, computational meaningfulness embodies the human capacity for rationality.

### Research results of behavioral studies should increase our understanding of human behavior in natural environments

When we take understanding human behavior in natural environments as a criterion to build a theory of behavior, it means that we are better able to describe, explain and predict human behavior (Gallistel, 2009, 2020; Yarkoni and Westfall, 2017; Jolly and Chang, 2019).

To better understand human behavior, as researchers we should leverage as natural stimuli and problems as possible in our experiments to capture realistic behavior. Despite the naturalness of stimuli in experiments lying along a spectrum, there can be described by three factors (Hamilton and Huth, 2020). First, a stimulus should represent a situation that a participant might reasonably be exposed to outside of an experimental setting. Second, the stimulus should appear in the same context as it would in real life. Third, the participants’ motivation and feeling to solve problems or make decisions should be as similar as possible in the experiments as in real life. These properties are reminiscent of previous requirements that psychologists should focus on the structure of natural environments that the mind relies on to perform inferences and to guide behavior (Brunswik, 1955; Simon, 1955; Todd and Gigerenzer, 2007; Holleman et al., 2020). We argue that these three factors are absent from typical BIASBEHA studies.

However, most current ecological studies have shown that we can bridge the gap between theoretically simple traditional psychological experimental setups and real-life human behavior. We describe these studies as follows. Generally, the effect of a stimulus or other message on people has been studied from the point of view of the recipient of the message. However, the expression of the original context by the person who conveys the message is also important for how the recipient understands the message. Whether it is a single message or an entire experiment setup, it oozes latent meaning that the receiver instinctively interprets (McKenzie and Nelson, 2003).

## Examples of studies that use natural stimuli in their experiments

The need for ecologically valid models has been also realized in the field of neuroscience (Nastase et al., 2020). As stated by Nastase (2021, 46): “We’re left with a veritable zoo of piecemeal models that are difficult to synthesize and, considered individually, account for a disappointing amount of variance under natural conditions.” Below we describe studies, which have used naturalistic and multidimensional stimuli in their experiments. Natural stimuli are videos, real advertisements, real health messages, stories, and immersive VR and AR technologies (Mobbs et al., 2021). Two groups of students participated in the Buzz study (Falk et al., 2013). A group of message communicators watched and evaluated new entertainment program concepts in the fMRI scanner intended for television. Immediately after the fMRI scan each message communicator presented the concepts outside of the scanner during video-interview. Then another group of students, who were message recipients, watched these videos. Finally, message recipients were asked how willing they were to recommend the concept proposals they saw to their friends. The study showed that successful ideas were associated with neural responses initially measured by fMRI in the mentalizing system and the reward system of message communicators when they first heard, before spreading them during video-interview. Similarly, message communicators more able to spread their preferences to others produced greater mentalizing system activity during initial encoding. Thus, people are very sensitive to the semantics of the messages and can interpret the intention of the sender (in this case message communicators), not only the literal meanings of these messages. It is also valuable that the results of the fMRI-experiment generalize beyond the experimental situation to the natural video interview and its viewing, as well as the personal preference caused by viewing.

Similarly, Falk et al. (2011, 2012) examined how smokers’ neurophysiological responses to antismoking advertisements predict subsequent smoking behavior. They found that the brain activation patterns in the valuation network of participants, when they were exposed to an anti-smoke message in the fMRI-scanner, more accurately predicted participants’ proclivity to quit smoking 1 month after the initial fMRI than traditional behavioral measurements. Even more noteworthy is that the activity in the same region of the mean brain activation patterns in the valuation network of participants predicted population-level behavior in response to health messages and provided information that was not conveyed by participants’ self-reports (Falk et al., 2012). Therefore, neural activity in the brain’s valuation network predicted the population response, whereas the self-report judgments did not. Thus, the participants’ neural patterns activation during fMRI-experiments “leaks” information about their valuation and desires, which have predictive power to real-like contexts.

In the same way, the research group of Genevsky and Knutson (2015); Genevsky et al. (2017) sought to find brain networks in laboratory samples to forecasted real microloans (Genevsky and Knutson, 2015) and crowdfund success (Genevsky et al., 2017) on the Internet. They found that the sample’s average activity in the part of the brain’s valuation network forecasted loan appeal and crowdfund success on the Internet. Findings demonstrate that a subset of the neural predictors in the valuation network of individual choice can generalize to forecast the market-level behavior of consumers.

### Naturalistic stimuli as the path toward novel findings in neurosciences

Heretofore we have argued that we humans are sensitive to meanings and semantics of the messages in contexts (Grice, 1975; Corner et al., 2010), not so much their literal content from a purely logical perspective, as the BIASBEHA-approach assumes. One of the pioneer researchers who used naturalistic context as stimuli is Uri Hasson. He has not so much looked for ways to predict people’s behavior outside of experimental situations, but rather he has tried to find a general common ground, especially for human communication and generally for human experiences. For example, in his seminal brain study (Hasson et al., 2004), the participants lay in a brain scanner and watched the Western film *The Good, the Bad, and the Ugly*. When the brain activations of all the participants measured by fMRI were looked at as a whole, the researchers found that the brains of the individuals activated in a very similar way to the important points of that classic Western movie. It was about the similar activation profile of individuals’ brains, i.e., synchronization in certain movie scenes. Especially emotionally powerful moments in the film synchronize the brains of the participants. Such emotional moments were stages that contained excitement, surprise, and joy. In addition, emotional activation also increased at points where the theme changed to another. Other researchers have found that scenes featuring people or animals generally and the other person’s eyes and face especially are especially powerful emotion stimulants and synchronize people’s brains in similar ways (Sharot and Garrett, 2016).

Hasson and colleagues have studied the basis of the human communication system and narrative processing in the brain (Lerner et al., 2011; Silbert et al., 2014; Yeshurun et al., 2021). The human communication system is an effective storyteller and it does record an individual’s memories, ideas, and dreams and transmits them to the brains of other people’s communication systems. Similarly, like watching a Western film, also when listening to a meaningful story, the participant’s brain showed similar activation patterns (i.e., synchronization) during the story listening. This occurred even when the same story was presented in Russian to subjects who were native speakers of Russia (Honey et al., 2012). Synchronization in higher-order brain regions, such as frontal, temporal, and parietal lobes, occurs regardless of the specific format of the narrative, e.g., textual or visual (Tikka et al., 2018). In other words, the meaning of the story (semantic structure) activates the human brain in similar ways even though the story is presented in a different syntax. More broadly, it is about a human’s capability to compute holistic meanings in their surroundings (=computational meaningfulness) and this process operates mostly based on meanings. However, BIASBEHA-approach operates almost exclusively at the level of stimulus forms and syntaxes.

Furthermore, Hasson and colleagues have found that the Default Mode Network (DMN) in the brain has an essential role on the individual level when an individual integrates extrinsic and intrinsic information and when s/he tries to establish shared meaning, communication tools, shared narratives, and social networks (Kauttonen et al., 2018; Yeshurun et al., 2021). DMN is usually considered an “intrinsic” region, specializing in internally oriented mental processes such as daydreaming, reminiscing, future planning, and creativity (Raichle et al., 2001; Heinonen et al., 2016). DMN with other brain networks together forms the comprehension system, which allows the formation of the meaning of the narrative on individual levels and allows it to couple across the speaker’s and listener’s minds during the production and comprehension of the same narrative. Nevertheless, this common ground for understanding breaks easily, when a certain part of the story is not understandable to the listener or if some part of the element does not belong in the story (Lerner et al., 2014; Yeshurun et al., 2017b). Elements that disturb the understanding of the story include, for example, scrambled sentences, nonsense sounds, and speaking sentences too quickly (Lerner et al., 2014). Even one unclear word can make it difficult to interpret the whole story (Zadbood et al., 2021).

Moreover, certain types of cultural products, such as stories, films, pieces of music, and speeches by well-known persons, cause the meaningful areas of people’s brains to activate in a very similar way (Schmälzle et al., 2015; Sharot and Garrett, 2016; Tikka et al., 2018; Zadbood et al., 2021). However, differences in people’s beliefs can substantially impact their interpretation of a series of events. When researchers manipulated participants’ beliefs in an fMRI study, this led two groups of participants to interpret the same narrative in different ways. They found that responses in the communication network of the brain tended to be similar among people who shared the same interpretation, but different from those of people with an opposing interpretation (Yeshurun et al., 2017b). This study showed that brain responses to the same narrative context tend to cluster together among people who share the same views. Similarly, small changes in the word of a story can lead to dramatically different interpretations of narratives among people despite the grammatical structure being similar across stories (Yeshurun et al., 2017a).

### Confirmation bias and framing effect as artifacts of impoverished experimental conditions

The brain studies described above give indications that human behavior is guided by the principle of meaningfulness. This sense-making process gives weight to certain features of the context at the expense of other features. The human brain combines incoming sensory information with prior intrinsic information—i.e. mental simulations in memory—to form rich, context-dependent models of contexts as they unfold over time (Yeshurun et al., 2021). The task of people’s brains is not to copy the physical world as accurately as possible via the senses but to support and participate in useful behaviors (Purves et al., 2015; Suomala, 2020; Suomala and Kauttonen, 2022).

Most previous studies of BIADBEHA literature assume discrete trials with no reference to participants’ real-life contexts. In addition, the experiments often are organized in ways, in which a subject chooses between only two options. In addition, these options are usually unfamiliar to participants and they cannot learn the meanings of these options. Therefore, the results according to the heuristics and biases framework relating to confirmation bias and framing effects give a too pessimistic picture of human behavior. When we take as a starting point the human ability to survive and adapt to countless life contexts, experiences of meaning and complexity enter the explanatory pattern. Some of the reason for this impoverished experimental tradition is a consequence of the fact that in the past it has been very difficult to study people in meaningful experimental settings. Today, the situation is different and as we described above, researchers can create real-like experiments, in which human participants could feel these situations are meaningful.

Previous examples showed, how it is possible to bring the multidimensionality of real contexts to brain studies and collect brain data in these situations in real time while the subject construct representations of contexts or solves various tasks in these experiments. In everyday life, a multitude of cognitive functions and the brain networks that subserve them are seamlessly and dynamically integrated (Snow and Culham, 2021). Rather than trying to isolate stimulus or task features, the idea of data-driven analysis strategies is that features that co-occur in the real world are likely jointly represented in brain organizational principles. When studying the fluctuations of human brain activations with fMRI—as previously described studies above—a huge amount of data is obtained from each subject. While the results based on this big data is sometimes difficult to interpret (i.e., difficult to explain the phenomenon behind the data), the benefits of enormous data from people’s brain are, that it can generalize to real-life situations and the ability to predict people’s choices in real-life situations (Knutson and Genevsky, 2018; Doré et al., 2019).

The term big data often refers to amounts of datasets that are enormous orders of magnitude larger than the datasets that behavioral scientists work with. In this case, data sets are sized terabytes or even petabytes in size (Yarkoni and Westfall, 2017). Similarly, the applications of big data have increased about people’s behavior. The possibility to access mobile and online data, coupled with a collect of enormous archival datasets from social networks and other websites, means that studies based on sample sizes of tens of thousands of participants (Schulz et al., 2019) to even sample sizes of millions of participants (Yarkoni and Westfall, 2017) is today possible. In addition to the fact that big data can be used to predict people’s future behavior (Knutson and Genevsky, 2018; Doré et al., 2019), its great advantage is that they provide a natural guard against overfitting (Yarkoni and Westfall, 2017; Hasson et al., 2020). The larger the data, the more representative it is of the population’s real behavior it is drawn from and it becomes increasingly difficult for a statistical model to capitalize on patterns that occur in the training data but not in the broader population (Yarkoni and Westfall, 2017). An essential challenge for this situation is how to analyze such enormous amounts of data. The development of machine learning algorithms gives tools to solve this challenge (Suomala and Kauttonen, 2022).

## Machine learning algorithms for analyzing multidimensional data relating to human behavior

How do the above complexity and multidimensionality affect designing and executing behavioral experiments? To describe, explain and predict human behavior better than before, it is useful to collect big datasets and analyze these data with data-driven methods and machine-learning algorithms. In recent years, machine learning has been able to solve difficult problems in many disciplines (Suomala and Kauttonen, 2022). Indeed, cognitive neuroscience is finally at a crossroads where we have enough data to start understanding brain-behavior associations (Zhou and Zuo, 2023). Together with increasing computational power and data set availability have led to breakthroughs in machine learning and artificial intelligence. Illustrative of this development is DeepMind’s program AlphaFold, which can predict the shape of almost all proteins based on their amino-acid sequences (Callaway, 2020). This problem has been biology’s grandest challenge for decades. Similar progress has been found in the context of geology (Beroza et al., 2021).

Machine learning algorithms allow researchers to fit large sets of parameters including both linear and non-linear functions and a goal state. When a large amount of data is given to these algorithms, they can find approximated functions that best explain the final result. In this way, for example, the amino acid chains associated with each protein pattern have been found. Machine learning is useful in understanding complex phenomena—like human behavior—in the following ways (Glaser et al., 2019; Suomala and Kauttonen, 2022). It helps to build better predictive models, identify predictive variables by applying regularization and finding causal relationships, benchmark linear and non-linear models, and serve as a model of the brain/mind to compare against algorithms. Due to the complexity of behavioral and neurophysiological datasets that can be both non-linear and recurrent, it is beneficial to apply machine learning methods that can extract meaningful dynamics and structures (Glaser et al., 2019).

The classical statistical modeling—which BIASBEHA uses almost exclusively—relies on inference rather than predictive power, and is insufficient when trying to find working principles of neurophysiology and behavior of humans (Yarkoni and Westfall, 2017; Jolly and Chang, 2019; Hasson et al., 2020). In a recent study by Schrimpf et al. (2021), researchers demonstrated that specific language models based on deep neural networks and transformer architecture could predict human neural and behavioral responses to linguistic input with perfect predictivity relative to the noise ceiling. The researcher suggests that “testing model ability to predict neural and behavioral measurements, dissecting the best-performing models to understand which components are critical for high brain predictivity, developing better models leveraging this knowledge, and collecting new data to challenge and constrain the future generations of neutrally plausible models of language processing” (Schrimpf et al., 2021). We argue that a similar approach should be pursued to other behavior as well beyond language. With enough data, artificial neural networks can handle the messy complexities of the natural world, including nonlinearities, redundancies, and interactions, as does the brain itself (Snow and Culham, 2021).

**Figure 1:**
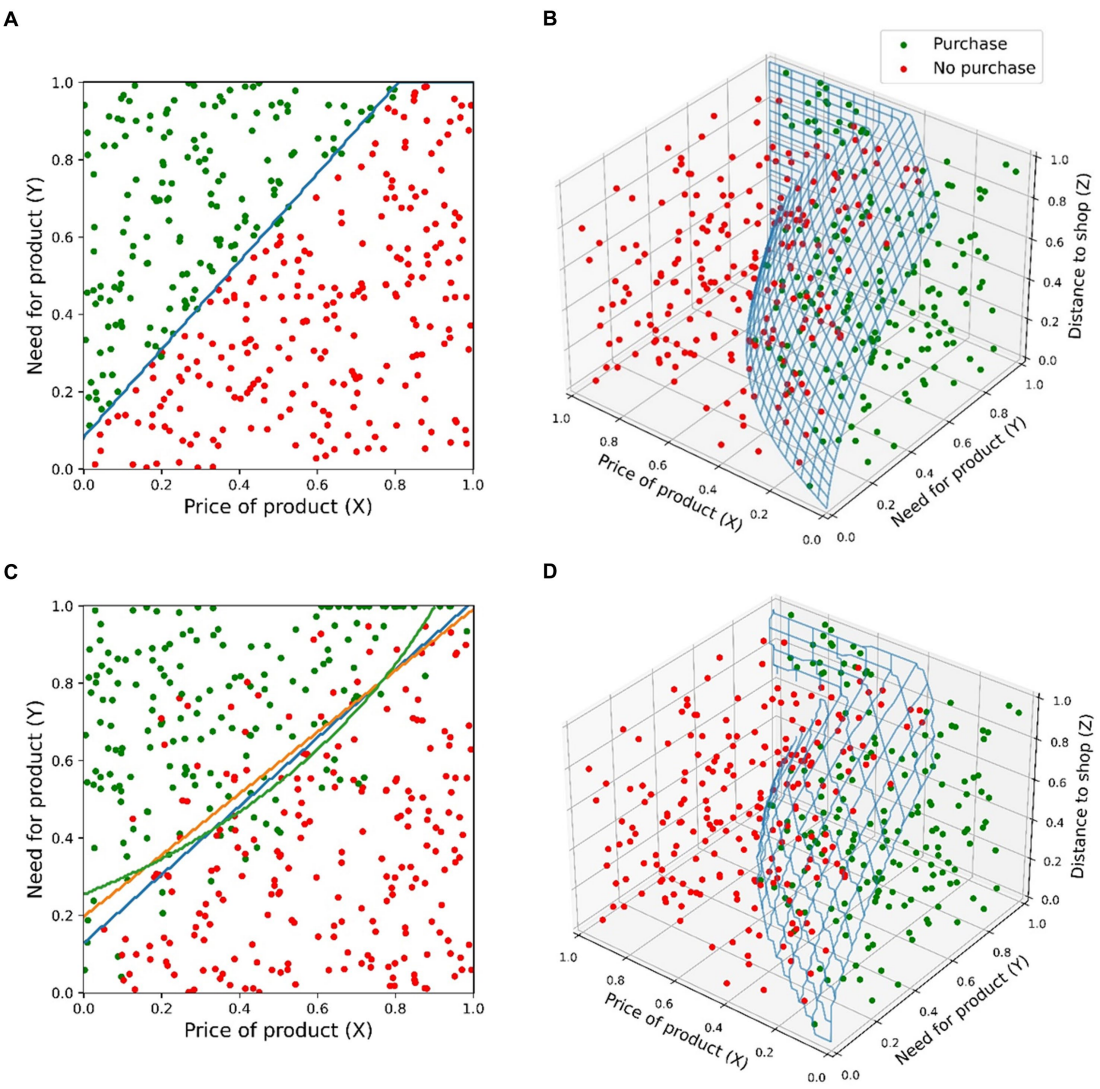
Hypothetical illustration of a decision to buy a certain product surveyed from 400 respondents. **(A)** Survey results in a laboratory setting depend on only two parameters: Price (X) and need (Y) for the product. Decision boundary fitted using a linear logistic regression model with red and green points corresponding to negative and positive decisions to buy. **(B)** A repeat of the experiment outside the laboratory with a third variable (Z) as a customer distance to the shop. The decision boundary is a complex, non-linear function. **(C)** Three models fitted to data with only two parameters included; models are linear (orange), quadratic (green), and neural network (blue). **(D)** Neural network model fitted to the full data with all three variables.

To make the discussion of impoverished experiments, irrational decisions, multidimensionality, and usefulness of machine learning techniques more concrete, let us consider an illustrative example of a hypothetical behavioral experiment. Imagine that an investigator wants to find out how the need and cost affect a decision to buy a certain product. The investigator asks 400 people how much they need this product (variable X) and whether they would buy the product at a specific price (variable Y). For simplicity, let us assume that these two variables are on an arbitrary scale between 0 (minimum value) and 1 (maximal value). The result is depicted in Figure 1A. The decision boundary appears clean and can be fitted well using a linear logistic regression model with 2 parameters. Using a typical 80–20 train-test data split (i.e., 80% for model training and 20% for testing), the error rate is 3.4%. Now, imagine another scenario where the same survey is performed by a brick-and-mortar shopkeeper, and the responders are expected to come by physically and buy the product. Now the physical distance between the shop and the customer (variable Z) will be a new variable. As depicted in Figure 1B, the decision boundary now appears as a non-linear function of the three variables. If this new data is plotted on X-Y plane, omitting Z, data appear noisy and some decisions irrational; even with a very high need for the product (close to 1) and very low product price (close to 0), some buying decisions are still negative and wise-versa. If we try to fit a model to this lower-dimensional data, results are poor as neither linear nor non-linear models work well. This is demonstrated in Figure 1C using linear (3 parameters) and quadratic (5 parameters) logistic regression models, and a neural network classifier model (3 hidden layers, 88 parameters). The models resulted in testing error rates 18.9%, 14.9%, and 14.9%. However, when all variables are included in the model, a good approximation of the original decision boundary can be found using a neural network model (98 parameters, error rate 0%) as shown in Figure 1D.

With the above example, we highlighted three aspects: context-dependent decision making, the difference between controlled (laboratory) experiments vs. messy complexities of real-life behavior, and the usefulness of machine learning and data-driven analysis favoring predictive power over model simplicity. In real-life scenarios, human decisions are affected by factors that are difficult to anticipate and emulate in impoverished, highly-controlled experimental settings. What may appear as irrational decisions in the second situation, are in reality rational when considering the constraints of real life, which in this case was the effort needed to buy the product. This highlights the importance of the multidimensional nature of ecological decision-making. Of course, our example is an oversimplification as a researcher cannot collect a dataset with all possible variables that could affect human behavior. However, this difficulty is not an excuse to omit ecological data collection completely.

As a summary, we may conclude that tightly-controlled (laboratory) experiments are useful for testing hypotheses about the contributions of components, e.g., which variables should be included in a model, ecological experiments are useful for testing whether those hypotheses generalize to natural settings, and for generating new hypotheses that consider the complexities of the organism in its environment (Nastase et al., 2020; Snow and Culham, 2021). Hypotheses should be formulated with ecological considerations in mind and rather than constraining data collection, data should be collected in representative contexts for the ecological behaviors that you want to study (Nastase et al., 2020).

## Summary and conclusion

The article describes typical BIASBEHA studies relating to confirmation bias and framing effects. Whereas these studies have shown that human reasoning differs decisively from the EUT’s concept of rationality, we presented a more realistic view of human rationality. We share the view of Gigerenzer (2018) to omit the ideas of irrationality and bias-centric view in behavioral economics, however, we need to take steps further toward life-like experimental settings and predictive modeling.

According to our approach, human is rational, because they can compute meaningful constellations and produce mental simulations of these, i.e., behave according to the principle of computational meaningfulness. Then rationality means firstly, that the human brain makes different contexts understandable by recognizing the relative importance of different elements in these contexts by optimizing multidimensional information relating to these contexts (Hofstadter, 1979; Hasson et al., 2020). Secondly, it means that a human can respond to contexts very flexibly and can make sense of ambiguous or contradictory messages. Third, it means that an individual can set complex goals and finally, it means that an individual can achieve these goals.

To understand human behavior and its multidimensionality, we need to study human behavior in real-life contexts. We presented some fMRI-studies, which have successfully shown, how using multidimensional data collected from real-like situations (by using videos, stories, real advertisements, and real health messages) can help our understanding and help to predict human behavior in real-life contexts. By using multidimensional stimuli and machine learning methodology we can go toward a better theory of human behavior. This means moving away from overly simplified, few-parameter models that generalize poorly with actual behavior and between subjects, and explaining behavior with a bias when decisions are meaningful from an individual’s point of view. One practical way to do this is to take advantage of immersive VR and AR technologies that allow building experiments closer to ecological conditions while also allowing experimental control.

Formalizing behavioral theories using neuroscientific and computational models provides a way to overcome the Flatland fallacy through the consideration of high-dimensional explanations of behavioral phenomena. Jolly and Chang (2019, p. 442) argue: “We believe the use of computational models will likewise better enable researchers to capture this complexity within psychological theories.” We agree and this article aims to sketch the theory of human behavior based on the principle of computational meaningfulness.

## Data availability statement

The original contributions presented in the study are included in the article/supplementary material, further inquiries can be directed to the corresponding author.

## Author contributions

All authors listed have made a substantial, direct, and intellectual contribution to the work and approved it for publication.

## Conflict of interest

The authors declare that the research was conducted in the absence of any commercial or financial relationships that could be construed as a potential conflict of interest.

## Publisher’s note

All claims expressed in this article are solely those of the authors and do not necessarily represent those of their affiliated organizations, or those of the publisher, the editors and the reviewers. Any product that may be evaluated in this article, or claim that may be made by its manufacturer, is not guaranteed or endorsed by the publisher.
